# The four pathways of assertiveness: a multidimensional framework for enhancing individual well-being

**DOI:** 10.3389/fpsyg.2025.1610807

**Published:** 2025-08-12

**Authors:** Naoki Yoshinaga, Scott Cooper

**Affiliations:** ^1^School of Nursing, Faculty of Medicine, University of Miyazaki, Miyazaki, Japan; ^2^SWA San Francisco, SWA Group, San Francisco, CA, United States

**Keywords:** assertiveness, subjective well-being, happiness, positive psychology, cognitive behavioral therapy, compassion, acceptance and commitment therapy

## Abstract

This paper introduces a broader theoretical framework for assertiveness that integrates traditional social assertiveness with three additional dimensions: behavioral, emotional, and mental assertiveness. We delineate four distinct yet interrelated pathways of assertiveness, applying common, non-technical terminology: “speaking up” (social), “jumping in” (behavioral), “embracing compassion” (emotional), and “accepting life” (mental). These dimensions are situated within an integrative framework aimed at enhancing individual well-being through intentional and context-sensitive agency. Drawing from existing psychological theories (e.g., grit, self-regulation, empathy), we clarify how each facet is differentiated from related constructs or theories. Furthermore, we outline practical directions for empirical testing, including candidate self-report measures, behavioral tasks, and qualitative assessments. The proposed model offers a multidimensional basis for future research and intervention development.

## 1 Introduction

In 1949, Andrew Salter, a pioneering behavioral psychologist, published *Conditioned Reflex Therapy* (Salter, 1949), in which he outlined specific techniques to help individuals reduce inhibition-based anxiety by learning to honestly express their thoughts and feelings—a process he called “feeling talk.” Although Salter’s book had limited initial impact on the field of psychology, it laid the foundation for assertiveness training as a component of behavioral psychology. His work influenced Joseph Wolpe, another prominent behavioral psychologist, who more formally conceptualized “assertion training” in the 1950s as a method for reducing anxiety (Wolpe, 1958). While Wolpe is best known for developing systematic desensitization and relaxation as primary methods for anxiety reduction, he also viewed self-assertiveness as an intentional behavioral intervention toward the same goal. Thus, from its inception, assertiveness was conceived as an intentional behavior aimed at promoting human well-being by reducing anxiety, with tools grounded in practical clinical psychology and a theoretical basis in behavioral psychology.

Since the 1950s, assertiveness has essentially been defined as the capacity to express one’s needs, rights, and opinions directly and respectfully, without infringing on the rights of others (Alberti and Emmons, 1970). Rooted in behavioral therapy, assertiveness training gained prominence in the 1970s (Rimm and Masters, 1974; Goldfried and Davison, 1976) and has since been widely adopted in clinical and educational settings. While this construct has proven valuable in supporting effective interpersonal communication in various contexts (e.g., Ames et al., 2017; Omura et al., 2017; Speed et al., 2018; Yoshinaga et al., 2018), it represents only one dimension of fully assertive living.

If we return to Wolpe’s original aim—assertiveness as intentional behavior that fosters well-being—modern developments in psychology have expanded our understanding of such intentional strategies. Over the past several decades, research has demonstrated that, in addition to assertive communication (i.e., “traditional assertiveness”), a variety of intentional behaviors derived from cognitive-behavioral therapy (CBT), including compassion- and acceptance-based approaches, also promote well-being.

The intent of the multidimensional model of assertiveness presented in this paper is to incorporate, under the single rubric of “assertiveness,” four intentional psychosocial strategies corresponding to four fundamental aspects of human functioning (Cooper and Yoshinaga, 2025). These strategies—articulated in accessible, non-technical terms—are “speaking up” (social assertiveness), “jumping in” (behavioral assertiveness), “embracing compassion” (emotional assertiveness), and “accepting life” (mental assertiveness). Table 1 defines these strategies and outlines the rationale for their inclusion, which is elaborated in the next section. Like traditional assertiveness, these behavioral strategies are derived from practical clinical psychology, but their theoretical foundation is based not solely on behavioral psychology, but on a broader, more integrative cognitive-behavioral framework that includes compassion- and acceptance-based elements.

**Table 1:** Principles of the four pathways of assertiveness and their impact on subjective well-being^a^.

Pathway	Principle	Impact on life satisfaction	Impact on positive and negative emotions
Speaking up (social assertiveness)	With speaking up, we assert ourselves by saying what we want and how we feel, even when our natural instinct might be to fight with or flee from others.	Life satisfaction means obtaining what we realistically need. This, in turn, requires direct, honest, and respectful communications.	Numerous studies confirm that social assertiveness training is helpful in reducing symptoms of socially generated anxiety and depression, as well as in improving relationships and measures of self-esteem (Speed et al., 2018).
Jumping in (behavioral assertiveness)	With jumping in, we assert ourselves by initiating actions we need or want to do, even when we do not feel like it.	Life satisfaction is defined as the degree to which we have what we want out of life. Life satisfaction is not possible without activating behaviors.	Several randomized controlled trials and meta-analyses provide evidence that behavioral activation (jumping into an activity) is as effective in dealing with depression as cognitive behavioral techniques and certain antidepressant drugs (e.g., Dimidjian et al., 2006; Mazzucchelli et al., 2009).
Embracing compassion (emotional assertiveness)	With compassion, we assert ourselves in responding to the suffering of others or ourselves, even when our natural instinct might be a lack of concern.	A major longitudinal study in Germany confirms that goals and activities involving altruism can result in significant long-term increases in life satisfaction (Headey et al., 2010). A growing body of research demonstrates that helping others is often associated with positive mental health outcomes for the helper (Brown and Brown, 2015).	Compassion itself can be a positive emotion and is connected to two other positive emotions: kindheartedness and sympathy. Research has demonstrated that people experience more positive emotions when spending money on others, rather than spending it on themselves (Dunn et al., 2014).
Accepting life (mental assertiveness)	With acceptance, we assert ourselves by taking things into perspective and softening judgments when confronted with the ups and downs of life, even when our natural instinct is to have negative judgments.	By accepting the realities of life with fewer negative judgments, our satisfaction with life markedly increases.	Acceptance is the foundation for contentment and love, two of the most beneficial positive emotions. Several researchers have found that greater acceptance can accompany less anxiety and depression. Acceptance entails softening negative judgments, which, in turn, can directly reduce negative emotions (e.g., Williams and Lynn, 2010).

a Excerpted and adapted from The Four Paths of Assertiveness (pp. 6–7, Table I.1), by Cooper and Yoshinaga (2025), Johns Hopkins University Press. Copyright 2025 by Johns Hopkins University Press. Reprinted with permission.

These strategies are not novel in themselves; they are well-established, evidence-based approaches with extensive clinical and research support. However, the multidimensional model that organizes them under the umbrella of assertiveness is novel. It offers an updated framework for assertive living that reflects insights from six decades of psychological research and clinical practice.

In our review of the literature, we have found no prior model that conceptualizes assertiveness in this multidimensional way. Traditional assertiveness has its roots in a singular behavioral tradition, whereas our model is embedded in cognitive-behavioral psychology, which is inherently multidimensional. That said, elements of behavioral, emotional, and cognitive flexibility are often present—if only implicitly—in traditional assertiveness. For example, behavioral assertiveness (i.e., behavioral activation) is both implied and necessary for speaking up. Respectfulness, a core aspect of traditional assertiveness, aligns with the kindness and perspective-taking of compassionate communication. Similarly, the flexibility found in techniques such as self-disclosure, shrugging, or using tentative phrasing (e.g., “maybe”) reflects the core principles of acceptance. Our model seeks to clarify and explicitly define these additional domains—behavioral, emotional, and mental—that support not only interpersonal functioning but also broader human well-being.

Although no empirical studies have yet examined this framework as a whole, there is ample research on each of the component strategies and their influence on well-being (see Table 1 for examples). For instance, studies have explored the relationship between traditional assertiveness and other intentional behavioral strategies central to our model, such as compassion (Hernández-Xumet et al., 2023).

While traditional assertiveness and compassion or acceptance are sometimes viewed as distinct or even conflicting strategies, this is not the case in the model we present. Speaking up, when practiced in isolation, can be ineffective, rigid, or even counterproductive if not integrated within a broader assertiveness framework that includes behavioral activation (action), compassion (kindness), and acceptance (flexibility). For example, one study found that both assertiveness and compassion were necessary for leaders to foster trust among their team members (Seco and Lopes, 2014). Within our multidimensional model, these strategies are united under the same conceptual umbrella. By applying precise definitions to each, we view them not as contradictory, but as complementary and mutually reinforcing.

**Figure 1:**
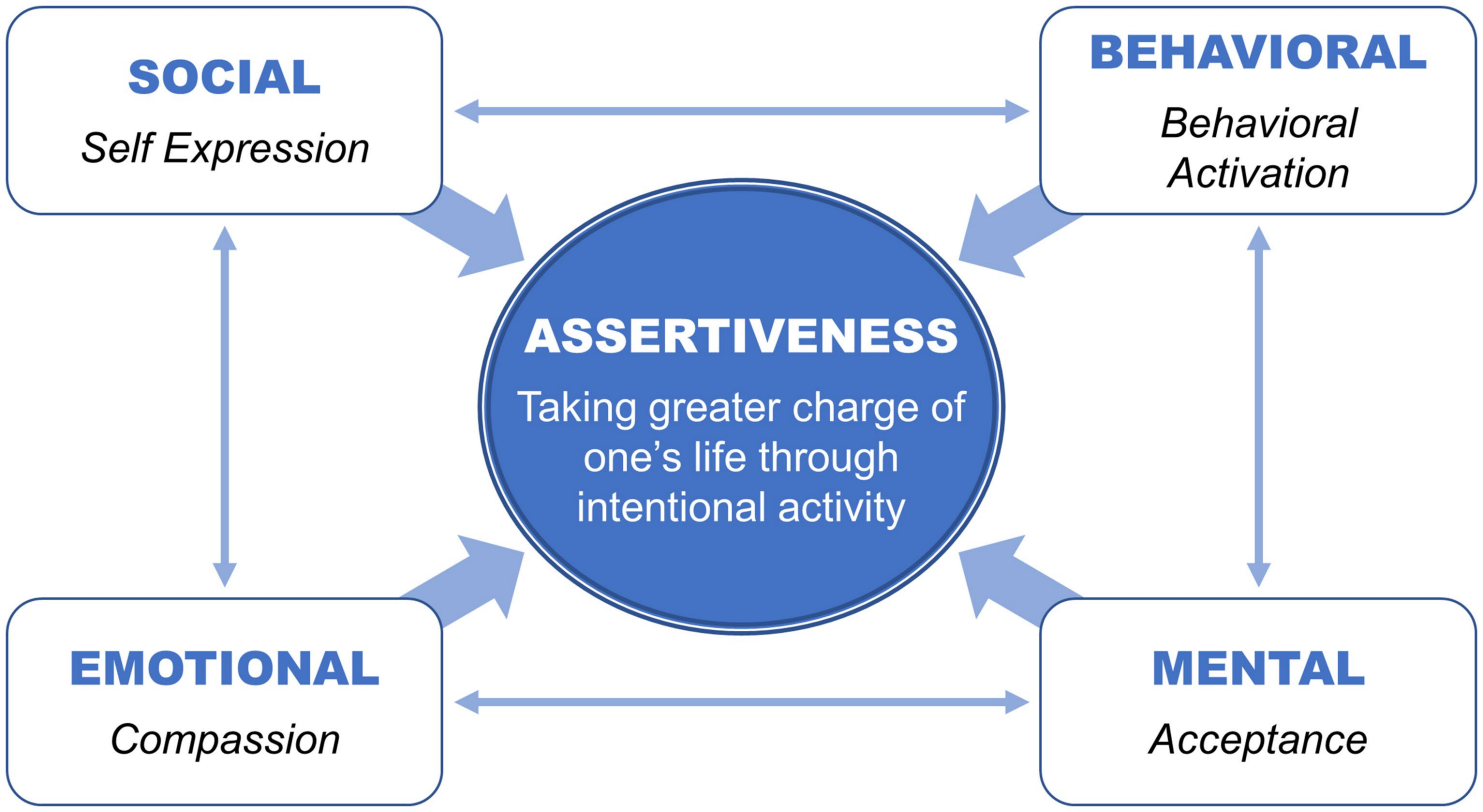
The four pathways of assertiveness (a more comprehensive, integrated model of assertiveness).

This article proposes an expanded theoretical model of assertiveness (see Figure 1), suggesting that subjective well-being is promoted through intentional engagement across four interdependent domains: social (“speaking up”), behavioral (“jumping in”), emotional (“embracing compassion”), and mental (“accepting life”). These four pathways are conceptualized as proactive strategies that help individuals not only enhance well-being but also navigate complex interpersonal, emotional, and existential challenges.

The central thesis proposed here is that a broader conceptualization of assertiveness entails not merely communicative competence, but the deliberate exercise of agency in shaping one’s behavior, emotional responses, and mindset in pursuit of a meaningful and fulfilling life (for more details, see Cooper and Yoshinaga, 2025). Rather than defining assertiveness solely in terms of self-expression, this model broadens the scope of assertiveness to include the proactive engagement of individuals in “taking greater charge of their lives” in pursuit of personal well-being and the well-being of others.

In the sections that follow, we first present the theoretical rationale for reconceptualizing assertiveness as a multidimensional construct grounded in intentional agency. We then define and explain each of the four proposed pathways—social, behavioral, emotional, and mental assertiveness—highlighting their conceptual distinctions from related constructs such as the Big Five personality traits, grit, self-regulation, empathy, and emotional intelligence. For each pathway, we describe empirically supported strategies for enhancing well-being and coping with everyday challenges. Finally, we discuss the practical and theoretical implications of our model, including potential applications in clinical and educational settings, suggested directions for empirical investigation, and guidance for operationalizing the model in future studies.

## 2 Theoretical foundations

The authors believe that traditional social assertiveness, when pursued solely for its own sake, may have limited value if it does not contribute to the enhancement of subjective well-being. Subjective well-being (or happiness) is generally conceptualized as comprising two key components: life satisfaction and the presence of positive emotions (and/or fewer negative emotions) (Diener and Biswas-Diener, 2008; Lyubomirsky, 2008). Empirical studies consistently affirm that life satisfaction and positive emotionality are both closely related to subjective well-being across diverse populations (Schütz et al., 2013; Berlin and Fors Connolly, 2019; Busseri and Erb, 2024). As suggested by Diener and Biswas-Diener (2008), an individual’s life satisfaction can be expressed as a function of the relationship between aspirations and attainments: Life Satisfaction = (What one wants) / (What one has). Accordingly, managing expectations and fostering contentment are as integral to well-being as the pursuit of goals.

Subjective well-being, also called “psychological wealth,” reflects the internal experiences that shape individuals’ perceived quality of life (Diener and Seligman, 2004; Diener and Biswas-Diener, 2008). While material conditions may provide the foundation for physical survival, psychological wealth plays a central role in fostering human flourishing (Ryff and Singer, 1998; Deci and Ryan, 2000). This perspective aligns with evolutionary and cognitive theories, which argue that although the body facilitates physical survival, it is the mind that interprets and gives meaning to human experiences (Baumeister, 1991; Cosmides and Tooby, 1992; Kahneman, 2011). Emotions, perceptions, and memories are filtered through cognitive faculties, underscoring the centrality of mental processes in shaping well-being. Ultimately, even the physical senses are not experienced until they are processed mentally.

Lyubomirsky and colleagues have identified three macro factors that determine human happiness (i.e., subjective well-being) (Lyubomirsky et al., 2005): (1) inherent genetic predispositions, (2) life circumstances, and (3) intentional activities. Existing evidence suggests that approximately 50 percent of subjective well-being is based on genetics, 10 percent on life circumstances, and 40 percent on intentional activities (Lyubomirsky et al., 2005). However, Lyubomirsky and colleagues subsequently re-examined this model and found that intentional activities can actually be quite variable in their impact on well-being (Lyubomirsky and Layous, 2013). This leaves intentional activities—what we assertively choose to do and think—as the lever we can use to make a difference in our well-being. Despite our genetic predispositions and life circumstances, we can impact our well-being through the intentional behaviors that we pursue. This intentional engagement in thought and action forms the essence of our broader conceptualization of assertive living.

The emotional component of subjective well-being encompasses both the frequency and intensity of positive affective states such as joy, gratitude, and serenity, alongside the regulation of negative emotions including fear, anger, and sadness (e.g., Diener and Larsen, 1993; Schimmack and Diener, 1997). The broaden-and-build theory posits that positive emotions expand cognitive and behavioral repertoires, thereby enhancing psychological resilience and social connectedness (Fredrickson, 2001). Conversely, persistent negative affect, unless regulated, may constrict thought-action tendencies and undermine adaptive functioning (Fredrickson and Levenson, 1998; McCracken et al., 2021).

Consequently, the expanded framework of assertiveness proposed herein is operationalized through four distinct yet interrelated domains of human experience—social, behavioral, emotional, and mental. These domains correspond to four assertive pathways: speaking up (social assertiveness), jumping in (behavioral assertiveness), embracing compassion (emotional assertiveness), and accepting life (mental assertiveness). Together, these pathways provide strategies for enhancing life satisfaction and positive affect, thereby contributing to individual well-being. Table 1 summarizes these four pathways, outlining their conceptual foundations and their contributions to subjective well-being.

While not intended as a direct theoretical foundation, the proposed four pathways of assertiveness can be heuristically linked to the evolution of CBTs (Hayes, 2019). The behavioral facet aligns with first-wave CBT’s emphasis on behavioral activation; the mental facet reflects second-wave CBT’s focus on cognitive restructuring; and the emotional facet shares common ground with third-wave approaches such as acceptance and commitment therapy. The social facet, though cross-cutting, aligns with all three waves in its emphasis on interpersonal functioning. These parallels are meant to illustrate the breadth of our model rather than to serve as its empirical basis.

To summarize, assertiveness—as conceptualized in this framework—involves the exercise of agency across interpersonal behavior, emotional regulation, actions, and thought processes. While distinct from subjective well-being as a construct, we hypothesize that engaging in assertive behavior across these domains may contribute to greater psychological well-being. This view is consistent with existing findings that assertiveness skills are positively associated with outcomes such as self-esteem, life satisfaction, and lower psychological distress (e.g., Zelenski et al., 2012; Speed et al., 2018).

In the following sections, each pathway will be explored in detail, alongside empirically supported strategies for promoting well-being and addressing everyday challenges.

## 3 Four pathways of assertiveness

### 3.1 Speaking up (social assertiveness)

#### 3.1.1 Definition and explanation

Social assertiveness, herein referred to as “speaking up,” has been traditionally conceptualized as the ability to directly and appropriately express one’s own needs, desires, and emotions, while simultaneously respecting the rights and perspectives of others (Alberti and Emmons, 1970). This interpersonal skill, in its various forms, has been recognized as a critical factor in enhancing individuals’ social functioning and subjective well-being (e.g., Sarkova et al., 2013; Ates, 2016; Carstensen and Klusmann, 2021). Typically, this traditional form of assertiveness has been understood as a preferred alternative to aggression (forceful, unfriendly conflict) and passivity (avoidance). However, as discussed below under the concept of “functional assertiveness,” social assertiveness can also be regarded as a communication continuum, where more aggressive or passive responses may be appropriate depending on the context—with a reasonable outcome being the standard of effectiveness. Moreover, expressions of assertiveness are shaped by cultural context. For example, communication styles perceived as appropriate in North America may be viewed as overly direct in East Asian or Latin American cultures (Park and Kim, 2008; Ma and Jaeger, 2010). The concept of functional assertiveness explicitly emphasizes context-appropriate expression over rigid stylistic norms.

Human emotional responses to challenging interpersonal situations frequently involve automatic and evolutionarily conserved fight-or-flight reactions. The perception of social threat often triggers affective responses, such as anger or fear, which may, in turn, elicit impulsive behavioral tendencies toward confrontation or avoidance (Blanchard and Blanchard, 2008). However, verbal assertiveness offers an alternative response strategy, characterized by deliberate and intentional communication governed by cognitive control rather than emotional reactivity (Parrott, 2001). Through the conscious enactment of assertive communication, individuals are able to override automatic defensive responses and instead employ verbal negotiation and problem-solving skills. Such assertive behavior facilitates the preservation of personal autonomy, while simultaneously promoting the development of constructive and mutually respectful interpersonal relationships. These interpersonal outcomes are considered to contribute to greater overall life satisfaction and psychological well-being (Sarkova et al., 2013; Ames et al., 2017).

Although social assertiveness may appear similar to the Big Five personality trait of extraversion, the two are conceptually distinct. Extraversion reflects a stable trait involving sociability and enthusiasm (McCrae and Costa, 1997; Ashton and Lee, 2005), while social assertiveness is a context-sensitive interpersonal skill that can be intentionally cultivated and flexibly applied (Speed et al., 2018). Assertive behavior can also be situationally activated in individuals with low trait extraversion (John et al., 2008; Speed et al., 2018), underscoring the learnable and volitional nature of assertiveness. Big Five traits may account for the portion of well-being shaped by genetic predispositions (Lyubomirsky et al., 2005; Lyubomirsky and Layous, 2013), whereas assertiveness relates to the portion of well-being influenced by intentional activity.

A growing body of empirical evidence has demonstrated the efficacy of assertiveness skills training in enhancing psychological adjustment and interpersonal functioning. Systematic reviews and meta-analyses have consistently shown that social skills training programs incorporating assertiveness components are associated with reductions in anxiety symptoms, particularly social anxiety, as well as improvements in self-esteem and decreases in depressive symptoms (Speed et al., 2018).

#### 3.1.2 Strategies for speaking up

Speaking up (social assertiveness) is enhanced through specific verbal and interpersonal skills that empower individuals to communicate their needs, set boundaries, and express their emotions constructively.

In the West, it has been common to emphasize a particular “style” of verbal assertiveness—typically characterized by direct and forceful communication and corresponding “assertive” body language. However, the concept of “functional assertiveness” emphasizes the importance of focusing on desired outcomes and using a style that is best suited to one’s personality, specific social situation, and culture (Mitamura, 2018). For example, a communication style that is considered normative in North America may be less effective—or even counterproductive—in Asia and Latin America (Ma and Jaeger, 2010). While a more aggressive, less friendly approach may be necessary in response to abusive or cruel behavior, a more friendly, persuasive, and respectful approach may be more effective in many everyday social situations. The key to “functional assertiveness” is focusing on the desired outcome, maintaining communication that is appropriate for the context, and demonstrating persistence—whether that persistence is friendly and polite or not.

Lazarus (1973) once identified four core abilities to help people be more socially effective: (1) openly expressing one’s desires and needs; (2) saying no; (3) expressing one’s positive and negative emotions; and (4) adequately beginning, maintaining, and ending conversations. Similar skills have been articulated by behavioral psychologists over time. In our review of existing tools, the following four core verbal skills have emerged as particularly useful in a broad variety of difficult social interactions:

1. “*I*-Statements” (“I think,” “I feel,” I want,” etc.) help individuals develop the habit of clearly and directly expressing honest thoughts and feelings. Andrew Salter’s introduction of *I*-statements (Salter, 1949) marked a pivotal moment in the development of assertiveness, although he did not specifically label this technique as “assertiveness training.”
2. “Saying No” is another simple yet sometimes difficult social skill that is foundational to social assertiveness. The pioneering assertiveness training book of the 1970s, *When I Say No, I Feel Guilty*, by Manuel Smith, was one of the first to articulate how to say no as a means of setting boundaries and practicing self-care (Smith, 1985).
3. “Simple Questions” refers to the skill of asking basic questions, even when one feels foolish doing so. While “*I*-Statements” help individuals express what they want and how they feel, learning to ask simple questions helps them more assertively seek what they want and need. This skill recognizes the right to acknowledge one’s limitations, including admitting when one does not know or fully understand something.
4. “Persistence” means not giving up until an acceptable outcome is achieved. The goal of social assertiveness in difficult situations is often self-determination and fair treatment, and in such situations, assertive communication is ineffective without persistence. Persistence entails skillfully continuing to pursue a desired resolution—or at least doing everything within one’s power to achieve a reasonable outcome. However, persistence should not be applied in potentially dangerous social situations.

Verbal assertiveness training is widely available through therapists, educational programs, and online platforms. Therapists can be particularly beneficial in cases where past trauma makes it difficult for individuals to express themselves assertively (Herman, 2015). Being the victim of past bullying, acute criticism, or other forms of mental or physical abuse can leave individuals with mental imagery and related emotional pain that inhibits assertive communication (Hackmann et al., 2000). Addressing underlying trauma issues first can support the development of assertive verbal skills. Professional interventions may include techniques such as imagery rescripting (Wild and Clark, 2011; Arntz, 2012).

### 3.2 Jumping in (behavioral assertiveness)

#### 3.2.1 Definition and explanation

We define behavioral assertiveness, referred to here as “jumping in,” as the act of initiating necessary or desired activities despite a lack of motivation. This plays a crucial role in overcoming obstacles such as natural instincts, unpleasant emotions, and social pressures. Often, individuals avoid engaging in activities that could bring satisfaction, leading to inactivity and negative emotions. In contrast, assertiveness involves “jumping in” to engage with chosen activities, which can positively influence emotions and behavior. Participating in enjoyable or productive activities has been shown to generate positive thoughts and improve mood, whereas excessive inactivity or negative interactions may contribute to negative emotions (Singh et al., 2023).

The concept of behavioral activation (i.e., jumping in)—a key component of cognitive-behavioral therapy—involves engaging in activities despite emotional resistance. This form of assertiveness has been demonstrated to be highly effective, particularly in addressing depression. Research indicates that behavioral activation, when intentionally practiced, can be more effective than purely cognitive strategies, such as countering negative thoughts (Mazzucchelli et al., 2009). A significant study by Dimidjian et al. (2006) found that behavioral activation is as effective as antidepressant medication (paroxetine) in treating depression.

In life, individuals must engage in both necessary and desired activities, such as fulfilling responsibilities and pursuing personal interests. While emotions, whether positive or negative, are part of life, they should not hinder action. Behavioral psychologists argue that when individuals face persistent sadness or depression, the natural response may be to withdraw, which can worsen the condition (Gross, 2002). Therefore, an active approach—engaging with others and seeking help—is recommended as an effective coping strategy for depression and negative emotional states (Coyne and DeLongis, 1986).

It is important to distinguish behavioral assertiveness from related constructs such as grit and self-control (Duckworth et al., 2007; Vohs and Baumeister, 2011). Behavioral assertiveness, as defined here, refers to the capacity to initiate action despite a lack of motivation or the presence of emotional resistance—particularly in situations characterized by avoidance tendencies. While grit emphasizes long-term perseverance toward distant goals and self-control involves inhibiting impulses, behavioral assertiveness focuses on the intentional initiation of present-moment action, especially in relation to daily obligations and personally valued activities. This definition aligns closely with the principles of behavioral activation, particularly in its emphasis on overcoming inertia arising from mood, avoidance, or discomfort in social situations.

#### 3.2.2 Strategies for jumping in

Jumping in (behavioral assertiveness) is supported by methods that promote goal-directed activity and help individuals overcome avoidance patterns. Below are three examples of practical strategies that can help activate behavior:

1. “Mental Rebuttals,” in line with other cognitive behavioral techniques, involve countering exaggerated thoughts such as “I cannot” or “It’s too hard,” which prevent us from initiating desired behavior. This is done by assertively applying reasonable and rational rebuttals to such thoughts to take the initial steps toward “jumping in.”
2. “Activity Scheduling” is a core technique in behavioral activation, typically involving the use of schedules, action lists, or calendars to plan daily activities (Jacobson et al., 2001; Lejuez et al., 2001; Martell et al., 2010).
3. “Values Clarification” involves intentionally identifying one’s values, documenting them, and pursuing life goals and activities aligned with those values. Values are shaped by unique personalities, skills, and aspirations, offering clarity and a personal compass for decision-making (Wilson et al., 2010).

### 3.3 Embracing compassion (emotional assertiveness)

#### 3.3.1 Definition and explanation

Emotional assertiveness, referred to here as “embracing compassion,” involves responding to suffering—both our own and that of others—with intentional, “assertive” acts of kindness and support. Suffering, in its various forms such as physical pain, emotional distress, anxiety, and grief, is an inherent aspect of the human experience. It can arise from external factors like illness, poverty, or life events, as well as from internal mental and emotional states. Regardless of its cause, suffering invariably involves a strong emotional component.

Our emotions and thoughts are deeply interconnected, influenced by biological, cultural, and psychological factors. Negative emotions are not solely triggered by immediate threats, but also by the mind’s ability to recall the past or imagine the future. For instance, remembering a loss can evoke grief, while worrying about potential challenges can lead to anxiety. Such emotional responses are a natural part of being human, as the mind constantly scans for both real and imagined dangers.

Compassion begins with recognizing the shared human experience of suffering and viewing others as fellow selves rather than objects. This perspective fosters a genuine concern for others’ well-being, even when their struggles are not fully known to us. While humans are driven by survival instincts, we also possess a natural inclination to help. Research shows that infants as young as 14–18 months engage in helping behaviors, such as comforting others and sharing, without external rewards (Hepach et al., 2012; Spinrad and Eisenberg, 2017). Moreover, adults who engage in altruistic behaviors, such as helping or volunteering, report increased life satisfaction and better physical and mental health outcomes (Headey et al., 2010; Brown and Brown, 2015). Ultimately, compassion and helping behaviors are essential components of human well-being.

Emotional assertiveness should be distinguished from related constructs such as empathy and emotional intelligence (Davis, 1994; Mayer et al., 2004). While empathy involves understanding or sharing another’s emotional state, and emotional intelligence refers to broader competencies like emotion recognition, regulation, and social navigation, emotional assertiveness emphasizes the intentional, compassionate expression of emotion—both inward and outward—especially in response to suffering. This includes not only caring behaviors toward others but also the practices of self-compassion and rational compassion. Rather than reflecting passive emotional sensitivity, emotional assertiveness is a proactive stance—a deliberate choice to respond to suffering (whether one’s own or others’) with kindness, support, and perspective-taking.

#### 3.3.2 Strategies for embracing compassion

Embracing compassion (emotional assertiveness) involves strategies that cultivate and express compassion as an intentional response to suffering. The following four strategies are examples of approaches that can cultivate more compassion in our lives.

1. “Rational Compassion,” as introduced by Bloom (2017), is an approach to compassion grounded in reason rather than pure emotion. Rational compassion includes seeing things from another’s point of view (perspective taking) and reasoning through the best course of helpful action, rather than depending upon empathy or sympathy as a motivator for helping others.
2. “Compassion Activation” is a form of behavioral activation focused on “jumping in” to help others, regardless of whether we feel like helping in the moment. Such efforts have been found to benefit the well-being of both the receiver and provider of help (Brown and Brown, 2015).
3. “Positive Communication” is helping others through kind and supportive words. Research highlights its importance in various contexts, such as in relationships and workplaces. For instance, Gottman (1994) suggests that healthy marriages require a 5-to-1 ratio of positive to negative communications, while high-performing organizational teams demonstrate a 6-to-1 ratio (Losada and Heaphy, 2004).
4. “Compassion-Based Meditation” is an effective method for cultivating compassionate attitudes and behaviors from within (Skwara et al., 2017). Loving-kindness meditation fosters feelings of kindness and acceptance toward oneself and others. Compassion meditation specifically focuses on cultivating care and concern for those experiencing suffering through mindful visualization and emotional engagement.

Self-compassion is a vital aspect of compassion-based practices (Neff, 2023). According to Neff (2011), self-compassion involves self-kindness, recognizing common humanity, and maintaining mindfulness. Practicing self-compassion allows individuals to respond to personal suffering with warmth and understanding rather than harsh self-criticism. Research indicates that self-compassion is associated with increased optimism, life satisfaction, and reduced depression and negative body image (Neff and Germer, 2013; Albertson et al., 2015). The four strategy examples above can also be applied to oneself.

### 3.4 Accepting life (mental assertiveness)

#### 3.4.1 Definition and explanation

Mental assertiveness, referred to here as “accepting life,” can be conceptualized as fostering greater acceptance of life by intentionally reducing unnecessary negative judgments. Excessive negative evaluations of oneself, others, or life circumstances are closely associated with dissatisfaction and negative emotions, two key components of human unhappiness. Acceptance refers to recognizing reality as it is, without denial or unrealistic expectations, especially regarding aspects that are beyond one’s control.

Previous research has demonstrated that cultivating acceptance through cognitive- and acceptance-based interventions contributes to improved mental health outcomes, particularly in managing anxiety symptoms (Butler et al., 2006; Gloster et al., 2020). Moreover, among older adults, greater acceptance of life experiences and associated unpleasant emotions has been linked to enhanced emotional well-being (Shallcross et al., 2013). Self-acceptance not only reduces anxiety but also fosters a greater acceptance of others (Falkenstein and Haaga, 2013).

Despite its benefits, acceptance is inherently challenging due to humans’ evolutionary tendency toward negativity bias. Negative judgments originally served as adaptive mechanisms for survival, drawing attention to potential threats (Rozin and Royzman, 2001).

Importantly, acceptance does not imply resignation or passive approval of adverse events. Rather, it involves intentionally observing experiences without excessive evaluation, reducing mental resistance, and acknowledging difficulties as inherent aspects of life. Acceptance can coexist with the appropriate use of necessary negative judgments, such as ethical discernment, problem-solving, and responding to injustice. Thus, fostering acceptance enables individuals to perceive life more accurately, reduce emotional turmoil, and engage in constructive actions to improve both personal well-being and the broader social environment.

#### 3.4.2 Strategies for accepting life

Accepting life (mental assertiveness) requires finding a more reasonable perspective and reducing unnecessary negative judgments. The following four strategies are examples of approaches that can be used to promote psychological flexibility and reduce maladaptive judgments.

1. “Perspective Shifting” involves reminding ourselves of “big idea” truths that provide important over-arching perspectives, e.g., “It’s part of life,” Humans aren’t perfect,” and “This just does not matter.” These macro ideas can provide quick perspective in moments when we experience a preponderance of negative assessments of life and people.
2. “Disputing Exaggerated Negative Thinking” is a key intervention in CBT for fostering cognitive flexibility (Leahy, 2006). Cognitive behavioral techniques emphasize noticing exaggerated, negative thoughts and intentionally considering alternative, more realistic perspectives that allow for greater acceptance of everyday events.
3. “Core Belief Reformation” involves critically examining and revising maladaptive beliefs that undermine reasonable acceptance of ourselves, others, and the nature of life (Ellis, 2000).
4. “Mindfulness Practice,” which has become widespread and well-known, is dedicated to developing greater non-judgmental acceptance in everyday living (Kabat-Zinn, 1994; Grossman et al., 2003), and can therefore contribute very directly to the objective of greater acceptance of life as an aspect of well-being.

One of the biggest challenges to psychological well-being is the impact of difficult, unpleasant emotions that arise. CBT—including behavioral activation—and antidepressant medication have been shown to be helpful in this regard (Dimidjian et al., 2006; Mazzucchelli et al., 2009). Another approach, promoted by Steven Hayes and others, is acceptance and commitment therapy (Hayes et al., 1999; Hayes, 2004). The emphasis of this therapy is to focus less on the content of thoughts and feelings and more on our relationship to those thoughts and feelings—applying less judgment and more acceptance and compassion toward painful mental activity that is difficult to change. Part of this approach emphasizes flexibly and nonjudgmentally observing mental activity as natural sensations, rather than focusing on the detailed content of such mental activity or assuming that such thoughts and emotions are “us” (Hayes et al., 1999; Hayes, 2004). Instead of avoiding or fighting such activity, one learns to take it in with less negative judgment and reaction. And quite importantly, one learns not to allow such sensations to inhibit commitment to, and engagement in valued daily activities.

Another major component of acceptance of life is self-acceptance. While self-acceptance overlaps with self-compassion, it is different. Self-compassion entails providing ourselves with kindness and support, while self-acceptance is a more fundamental belief about the self. It is holding the conviction that one is a regular, worthwhile human being regardless of mistakes and limitations. We unconditionally accept ourselves, just as we unconditionally accept a son or daughter or best friend. Self-acceptance does not mean that everything we do is good, or that we like everything we do. It cannot be an excuse for cruel or harmful behavior. We still need to take responsibility for our actions. But it does mean that we accept ourselves as regular, imperfect human beings through the thick and thin of everyday living.

Radical acceptance, a concept popularized by Brach (2003), builds on mindfulness by encouraging acceptance of life’s realities—our thoughts, emotions, and external circumstances—without judgment or the need for control. This practice invites individuals to let go of mental resistance to things beyond their control, fostering compassion and mental clarity. In mindfulness meditation, moments of “radical acceptance” occur when we let go of mental turmoil and embrace life as it is, allowing the universe (or a higher power) to unfold as it will (Brach, 2003). Both mindfulness and radical acceptance work together to enhance mental well-being and promote a greater awareness of life’s present moment.

## 4 Discussion

### 4.1 Limitations of the proposed model

As a theoretical framework, the four-pathway model of assertiveness has several limitations. First, the model remains conceptual and has yet to be tested empirically as an integrated structure. While the components are grounded in evidence-based psychological strategies, their interrelationships and distinctiveness require further empirical examination. Second, the model’s broad scope poses challenges for precise operationalization, especially with respect to mental and emotional assertiveness, which may overlap with constructs such as psychological flexibility and compassion. Third, although we have attempted to define each pathway with theoretical clarity, future studies are needed to establish discriminant validity relative to existing constructs. Finally, the model’s cross-cultural applicability remains uncertain; the salience and appropriateness of each assertiveness pathway may vary substantially across cultural contexts, requiring culturally sensitive adaptations and comparative investigations. These limitations should be addressed in future conceptual refinement and empirical validation.

### 4.2 Implications for practice and future research

#### 4.2.1 Directions for future research

Empirical investigation of the four-pathway model of assertiveness is warranted to evaluate its predictive validity, clinical utility, and cross-cultural generalizability.

In addition, future research should clarify the model’s testability by explicitly addressing its underlying assumptions. If assertiveness is conceptualized as a state rather than a stable trait, longitudinal designs will be essential to capture its dynamic nature over time and in response to interventions. Such studies could explore intraindividual variability in assertiveness across contexts or life phases. Moreover, if the model implies normative benchmarks for adaptive assertiveness, comparative studies across populations (e.g., clinical vs. non-clinical groups, cultural or occupational samples) will be necessary to assess its generalizability and contextual appropriateness. These lines of inquiry may help determine whether certain assertiveness pathways are more salient or effective depending on situational demands or individual characteristics.

Future research should focus on the following:

- Development and validation of psychometric instruments to assess the four hypothesized dimensions of assertiveness (social, behavioral, emotional, and mental).
- Experimental and longitudinal investigations comparing the differential and combined effects of pathway-specific interventions on psychosocial outcomes, such as well-being, self-esteem, and interpersonal functioning.
- Examination of mediating and moderating mechanisms, including self-efficacy, emotional regulation, and value congruence, that may underlie the link between assertiveness and subjective well-being (Bandura, 1997).
- Lifespan and clinical population studies, including applications in adolescents, older adults, and individuals with mood or anxiety disorders, to examine the model’s relevance across developmental and diagnostic spectra.

In addition, theoretically, the model invites further exploration of assertiveness as an integrated construct within the broader frameworks of psychological flexibility, eudaimonic well-being, and self-determination theory (Hayes et al., 1999; Ryan and Deci, 2000). Future studies should also examine the convergent and discriminant validity of each pathway. While each is defined with distinct theoretical boundaries, empirical studies are needed to establish their uniqueness relative to related constructs—for example:

- Social assertiveness vs. extraversion or communication competence
- Behavioral assertiveness vs. grit or self-control
- Emotional assertiveness vs. empathy or emotional intelligence
- Mental assertiveness vs. cognitive flexibility or acceptance

Establishing construct validity through factor analysis and correlations with existing measures will be essential in refining the model’s empirical value.

Although substantial research supports the effectiveness of each behavioral strategy included in this model, additional studies could examine the interrelationships among these strategies and their combined impact on well-being. For instance, it is plausible that speaking up (traditional assertiveness) and jumping in (behavioral activation) are highly interrelated, as the former depends on the latter. Future research could also explore the connections between assertive self-expression and compassion, assertive self-expression and acceptance, and between compassion and acceptance. While these relationships may seem intuitive, exploratory research using surveys and qualitative interviews could offer empirical clarification.

Additionally, the model raises questions about the cultural specificity of assertive behaviors. For instance, in collectivist societies, “speaking up” may be less socially rewarded than “embracing compassion” or “accepting life” (Park and Kim, 2008; Gelfand et al., 2011). Future studies might explore the cultural modulation of the relative salience and effectiveness of each pathway across diverse sociocultural contexts.

By offering a broader conceptualization of assertiveness, this model contributes to ongoing efforts to refine psychological interventions that support sustainable well-being and personal growth. Its multidimensional nature aligns with contemporary movements in integrative psychotherapy and transdiagnostic approaches (Barlow et al., 2004; Norcross and Goldfried, 2005), offering a promising avenue for both scholarly inquiry and practical implementation. Its flexibility also makes it amenable to digital health delivery and scalable interventions across diverse populations.

#### 4.2.2 Practical implications for empirical testing

To complement the theoretical directions outlined above, we propose preliminary ideas for how each of the four pathways of assertiveness might be operationalized in empirical research. These suggestions are intended not as finalized measures, but as conceptual starting points to guide future measurement development and validation studies.

Social assertiveness may be assessed using structured role-play tasks or interpersonal simulations in which participants navigate challenging social exchanges (e.g., refusing unreasonable demands, giving constructive criticism). Behavioral coding systems could be employed to evaluate the clarity, calmness, and respectfulness of assertive responses (Bolger et al., 2003; Shiffman et al., 2008; Iida et al., 2012). Additionally, self-report measures may capture perceived confidence and discomfort in boundary-setting situations, while observer (e.g., peer or supervisor) ratings could serve as external indicators of socially assertive behavior.

Behavioral assertiveness may be measured by the frequency and quality of proactive behaviors that reflect personal goals, particularly in situations involving interpersonal challenge or judgment. Daily diary methods and ecological momentary assessments could track such actions in real time (Bolger et al., 2003; Shiffman et al., 2008; Iida et al., 2012), including decisions to speak up in meetings, pursue goals despite criticism, or act against group pressure. Task-based paradigms (e.g., assertive action tasks in virtual environments) may also offer standardized behavioral indices of this construct.

Emotional assertiveness could be operationalized as the intentional enactment of compassion toward oneself and others in response to suffering. This may be assessed through self-report measures capturing frequency and motivation behind helping behaviors, expressions of support, and compassionate communication. Observational or informant-based assessments of prosocial and self-compassionate behavior may complement self-report data. Additionally, participation in compassion-based practices (e.g., loving-kindness or self-compassion meditation) could be examined as behavioral indicators of emotional assertiveness (Jazaieri et al., 2013).

Mental assertiveness reflects an internal stance of self-directed agency in engaging with one’s thoughts, beliefs, and values. It involves not only cognitive flexibility and self-reflection, but also the capacity to accept uncomfortable inner experiences without avoidance or suppression. This construct may be assessed through instruments that capture adaptive inner dialogue, value-guided decision-making, and openness to internal discomfort—concepts closely related to psychological flexibility (Kashdan and Rottenberg, 2010). Performance-based tasks assessing cognitive flexibility (e.g., perspective-shifting, identifying core personal values) and response style coding of open-ended responses may offer behavioral indicators. In addition, self-report measures derived from acceptance- and commitment-based frameworks—such as the Acceptance and Action Questionnaire (Bond et al., 2011)—could provide valuable indices of mental assertiveness as acceptance in action (Hayes et al., 2012). Qualitative methods may further reveal how individuals engage with internal barriers in the service of meaningful life directions.

These proposed operationalizations aim to facilitate the design of future studies examining the psychometric structure, predictive validity, and clinical relevance of each assertiveness pathway. Employing a combination of quantitative and qualitative methods across diverse populations will be important for building a comprehensive empirical foundation for the model.

#### 4.2.3 Clinical and educational applications

The four-pathway model of assertiveness may also inform the development of integrated therapeutic programs that emphasize assertiveness not only as a social skill but also as a holistic psychological orientation encompassing behavior, emotion, and mental. Cognitive-behavioral therapists, health psychologists, and educators could employ the four-pathway framework to assist clients and students in identifying and addressing areas of under-engagement across these domains.

Furthermore, the model’s intelligibility and cross-domain relevance make it a suitable foundation for psychoeducational materials and self-help interventions. Structured programs based on this framework could be delivered in clinical, community, or workplace settings to enhance resilience, foster self-determination, and improve quality of life (Speed et al., 2018). Assertiveness training protocols could be updated to include exercises in behavioral activation, self-compassion, and mindfulness-based acceptance practices (Harris, 2019), thus moving beyond traditional social skills training toward more comprehensive models of psychological flexibility.

In educational settings, this model can be incorporated into school-based social–emotional learning (SEL) curricula, university counseling programs, and professional training for caregivers and educators. Teaching young people and emerging adults to “jump in” or “accept life” may be especially effective for promoting autonomy and emotional regulation during transitional life stages.

This model may serve as a foundation for a new generation of assertiveness-based interventions that extend beyond traditional social skills training by incorporating behavioral, emotional, and mental dimensions.

## 5 Conclusion

This paper has proposed a multidimensional framework of assertiveness encompassing social, behavioral, emotional, and mental facets. By clearly distinguishing each pathway from related constructs, we aimed to advance theoretical clarity and offer a more nuanced lens through which assertiveness can be both understood and cultivated.

Our model highlights the importance of intentional, context-sensitive agency in promoting psychological well-being. Importantly, we have offered concrete proposals for operationalizing and empirically validating each dimension, thereby linking conceptual innovation with applied research.

Future studies should explore the distinct and interactive effects of these pathways across different cultures and populations, and examine their implications for education, psychotherapy, and health promotion.

## Data Availability

The original contributions presented in the study are included in the article/supplementary material, further inquiries can be directed to the corresponding author.

## References

[ref1] Alberti R. E. Emmons M. L. (1970). Your perfect right: A guide to assertive behavior. Oxford: Impact Press.

[ref2] Albertson E. R. Neff K. D. Dill-Shackleford K. E. (2015). Self-compassion and body dissatisfaction in women: a randomized controlled trial of a brief meditation intervention. Mindfulness 6, 444–454. doi: 10.1007/s12671-014-0277-3

[ref3] Ames D. Lee A. Wazlawek A. (2017). Interpersonal assertiveness: inside the balancing act. Soc. Personal. Psychol. Compass 11:e12317. doi: 10.1111/spc3.12317

[ref4] Arntz A. (2012). Imagery descripting as a therapeutic technique: review of clinical trials, basic studies, and research agenda. J. Exp. Psychopathol. 3, 189–208. doi: 10.5127/jep.024211

[ref5] Ashton M. C. Lee K. (2005). Honesty-humility, the big five, and the five-factor model. J. Pers. 73, 1321–1354. doi: 10.1111/j.1467-6494.2005.00351.x, PMID: 16138875

[ref6] Ates B. (2016). Perceived social support and assertiveness as a predictor of candidates psychological counselors’ psychological well-being. Int. Educ. Stud. 9, 28–39. doi: 10.5539/ies.v9n5p28

[ref7] Bandura A. (1997). Self-efficacy: The exercise of control. New York: W H Freeman/Times Books/Henry Holt & Co.

[ref8] Barlow D. H. Allen L. B. Choate M. L. (2004). Toward a unified treatment for emotional disorders. Behav. Ther. 35, 205–230. doi: 10.1016/S0005-7894(04)80036-4 27993336

[ref9] Baumeister R. F. (1991). Meanings of life. New York: Guilford Press.

[ref10] Berlin M. Fors Connolly F. (2019). The association between life satisfaction and affective well-being. J. Econ. Psychol. 73, 34–51. doi: 10.1016/j.joep.2019.04.010

[ref11] Blanchard D. C. Blanchard R. J. (2008). “Defensive behaviors, fear, and anxiety” in Handbook of anxiety and fear (San Diego: Elsevier Academic Press), 63–79.

[ref12] Bloom P. (2017). Against empathy: The case for rational compassion. New York: HarperCollins.

[ref13] Bolger N. Davis A. Rafaeli E. (2003). Diary methods: capturing life as it is lived. Annu. Rev. Psychol. 54, 579–616. doi: 10.1146/annurev.psych.54.101601.145030, PMID: 12499517

[ref14] Bond F. W. Hayes S. C. Baer R. A. Carpenter K. M. Guenole N. Orcutt H. K.. (2011). Preliminary psychometric properties of the acceptance and action questionnaire–II: a revised measure of psychological inflexibility and experiential avoidance. Behav. Ther. 42, 676–688. doi: 10.1016/j.beth.2011.03.007, PMID: 22035996

[ref15] Brach T. (2003). Radical acceptance: Embracing your life with the heart of a Buddha. New York: Bantam Books.

[ref16] Brown S. L. Brown R. M. (2015). Connecting prosocial behavior to improved physical health: contributions from the neurobiology of parenting. Neurosci. Biobehav. Rev. 55, 1–17. doi: 10.1016/j.neubiorev.2015.04.004, PMID: 25907371

[ref17] Busseri M. A. Erb E. M. (2024). The happy personality revisited: re-examining associations between big five personality traits and subjective well-being using meta-analytic structural equation modeling. J. Pers. 92, 968–984. doi: 10.1111/jopy.12862, PMID: 37462061

[ref18] Butler A. C. Chapman J. E. Forman E. M. Beck A. T. (2006). The empirical status of cognitive-behavioral therapy: a review of meta-analyses. Clin. Psychol. Rev. 26, 17–31. doi: 10.1016/j.cpr.2005.07.003, PMID: 16199119

[ref19] Carstensen B. Klusmann U. (2021). Assertiveness and adaptation: prospective teachers’ social competence development and its significance for occupational well-being. Br. J. Educ. Psychol. 91, 500–526. doi: 10.1111/bjep.12377, PMID: 32914428

[ref20] Cooper S. Yoshinaga N. (2025). The four paths of assertiveness: Speaking up, jumping in, embracing compassion, and accepting life. Baltimore: Johns Hopkins University Press.

[ref21] Cosmides L. Tooby J. (1992). “Cognitive adaptations for social exchange” in The adapted mind: Evolutionary psychology and the generation of culture. eds. Barkow J. H. Cosmides L. Tooby J.. (New York: Oxford University Press), 163–228.

[ref22] Coyne J. C. DeLongis A. (1986). Going beyond social support: the role of social relationships in adaptation. J. Consult. Clin. Psychol. 54, 454–460. doi: 10.1037/0022-006X.54.4.454, PMID: 3745597

[ref23] Davis M. H. (1994). Empathy: A social psychological approach. Boulder: Westview Press.

[ref24] Deci E. L. Ryan R. M. (2000). The “what” and “why” of goal pursuits: human needs and the self-determination of behavior. Psychol. Inq. 11, 227–268. doi: 10.1207/S15327965PLI1104_01

[ref25] Diener E. Biswas-Diener R. (2008). Happiness: Unlocking the mysteries of psychological wealth. Malden: Blackwell Publishing.

[ref26] Diener E. Larsen R. J. (1993). “The experience of emotional well-being” in Handbook of emotions. eds. Lewis M. Haviland J. M.. (New York: The Guilford Press), 405–415.

[ref27] Diener E. Seligman M. E. P. (2004). Beyond money: toward an economy of well-being. Psychol. Sci. Public Interest 5, 1–31. doi: 10.1111/j.0963-7214.2004.00501001.x 26158992

[ref28] Dimidjian S. Hollon S. D. Dobson K. S. Schmaling K. B. Kohlenberg R. J. Addis M. E.. (2006). Randomized trial of behavioral activation, cognitive therapy, and antidepressant medication in the acute treatment of adults with major depression. J. Consult. Clin. Psychol. 74, 658–670. doi: 10.1037/0022-006X.74.4.658, PMID: 16881773

[ref29] Duckworth A. L. Peterson C. Matthews M. D. Kelly D. R. (2007). Grit: perseverance and passion for long-term goals. J. Pers. Soc. Psychol. 92, 1087–1101. doi: 10.1037/0022-3514.92.6.1087, PMID: 17547490

[ref30] Dunn E. W. Aknin L. B. Norton M. I. (2014). Prosocial spending and happiness: using money to benefit others pays off. Curr. Dir. Psychol. Sci. 23, 41–47. doi: 10.1177/0963721413512503

[ref31] Ellis A. (2000). How to control your anxiety before it controls you. New York: Citadel.

[ref32] Falkenstein M. J. Haaga D. A. F. (2013). “Measuring and characterizing unconditional self-acceptance” in The strength of self-acceptance: Theory, practice and research. ed. Bernard M. E.. (New York: Springer New York), 139–151.

[ref33] Fredrickson B. L. (2001). The role of positive emotions in positive psychology: the broaden-and-build theory of positive emotions. Am. Psychol. 56, 218–226. doi: 10.1037/0003-066X.56.3.218 11315248 PMC3122271

[ref34] Fredrickson B. L. Levenson R. W. (1998). Positive emotions speed recovery from the cardiovascular sequelae of negative emotions. Cogn. Emot. 12, 191–220. doi: 10.1080/026999398379718, PMID: 21852890 PMC3156608

[ref35] Gelfand M. J. Raver J. L. Nishii L. Leslie L. M. Lun J. Lim B. C.. (2011). Differences between tight and loose cultures: a 33-nation study. Science 332, 1100–1104. doi: 10.1126/science.1197754, PMID: 21617077

[ref36] Gloster A. T. Walder N. Levin M. E. Twohig M. P. Karekla M. (2020). The empirical status of acceptance and commitment therapy: a review of meta-analyses. J. Contextual Behav. Sci. 18, 181–192. doi: 10.1016/j.jcbs.2020.09.009

[ref37] Goldfried M. R. Davison G. C. (1976). Clinical behavior therapy. New York: Holt, Rinehart & Winston.

[ref38] Gottman J. M. (1994). What predicts divorce? The relationship between marital processes and marital outcomes. Hillsdale: Lawrence Erlbaum Associates, Inc.

[ref39] Gross J. J. (2002). Emotion regulation: affective, cognitive, and social consequences. Psychophysiology 39, 281–291. doi: 10.1017/S0048577201393198, PMID: 12212647

[ref40] Grossman P. Niemann L. Schmidt S. Walach H. (2003). Mindfulness-based stress reduction and health benefits: a meta-analysis. Focus. Altern. Complement. Ther. 8:500. doi: 10.1111/j.2042-7166.2003.tb04008.x 15256293

[ref41] Hackmann A. Clark D. M. McManus F. (2000). Recurrent images and early memories in social phobia. Behav. Res. Ther. 38, 601–610. doi: 10.1016/S0005-7967(99)00161-8, PMID: 10846808

[ref42] Harris R. (2019). ACT made simple: An easy-to-read primer on acceptance and commitment therapy. Oakland: New Harbinger Publications.

[ref43] Hayes S. C. (2004). Acceptance and commitment therapy, relational frame theory, and the third wave of behavioral and cognitive therapies. Behav. Ther. 35, 639–665. doi: 10.1016/S0005-7894(04)80013-3 27993338

[ref44] Hayes S. C. (2019). A liberated mind: How to pivot toward what matters. New York: Avery.

[ref45] Hayes S. C. Strosahl K. D. Wilson K. G. (1999). Acceptance and commitment therapy: An experiential approach to behavior change. New York: Guilford Press.

[ref9001] Hayes S. C. Strosahl K. D. Wilson K. G. (2012). Acceptance and commitment therapy: The process and practice of mindful change, 2nd ed. New York: Guilford Press.

[ref46] Headey B. Muffels R. Wagner G. G. (2010). Long-running German panel survey shows that personal and economic choices, not just genes, matter for happiness. PNAS 107, 17922–17926. doi: 10.1073/pnas.1008612107, PMID: 20921399 PMC2964245

[ref47] Hepach R. Vaish A. Tomasello M. (2012). Young children are intrinsically motivated to see others helped. Psychol. Sci. 23, 967–972. doi: 10.1177/0956797612440571, PMID: 22851443

[ref48] Herman J. (2015). Trauma and recovery: The aftermath of violence—From domestic abuse to political terror. New York: Basic Books.

[ref49] Hernández-Xumet J.-E. García-Hernández A.-M. Fernández-González J.-P. Marrero-González C.-M. (2023). Beyond scientific and technical training: assessing the relevance of empathy and assertiveness in future physiotherapists: a cross-sectional study. Health Sci. Rep. 6:e1600. doi: 10.1002/hsr2.1600, PMID: 37799443 PMC10547931

[ref50] Iida M. Shrout P. E. Laurenceau J.-P. Bolger N. (2012). “Using diary methods in psychological research” in APA handbook of research methods in psychology, Vol 1: Foundations, planning, measures, and psychometrics. eds. Cooper H. Camic P. M. Long D. L. Panter A. T. Rindskopf D. Sher K. J. (Washington: American Psychological Association), 277–305.

[ref51] Jacobson N. S. Martell C. R. Dimidjian S. (2001). Behavioral activation treatment for depression: returning to contextual roots. Clin. Psychol. Sci. Pract. 8, 255–270. doi: 10.1093/clipsy.8.3.255

[ref52] Jazaieri H. Jinpa G. T. McGonigal K. Rosenberg E. L. Finkelstein J. Simon-Thomas E.. (2013). Enhancing compassion: a randomized controlled trial of a compassion cultivation training program. J. Happiness Stud. 14, 1113–1126. doi: 10.1007/s10902-012-9373-z

[ref53] John O. P. Robins R. W. Pervin L. A. (2008). Handbook of personality: Theory and research. New York: The Guilford Press.

[ref54] Kabat-Zinn J. (1994). Wherever you go, there you are: Mindfulness meditation in everyday life. New York: Hyperion.

[ref55] Kahneman D. (2011). Thinking, fast and slow. New York: Farrar, Straus and Giroux.

[ref56] Kashdan T. B. Rottenberg J. (2010). Psychological flexibility as a fundamental aspect of health. Clin. Psychol. Rev. 30, 865–878. doi: 10.1016/j.cpr.2010.03.001, PMID: 21151705 PMC2998793

[ref57] Lazarus A. A. (1973). On assertive behavior: a brief note. Behav. Ther. 4, 697–699. doi: 10.1016/S0005-7894(73)80161-3

[ref58] Leahy R. L. (2006). The worry cure: Seven steps to stop worry from stopping you. New York: Harmony Books.

[ref59] Lejuez C. W. Hopko D. R. Hopko S. D. (2001). A brief behavioral activation treatment for depression: treatment manual. Behav. Modif. 25, 255–286. doi: 10.1177/0145445501252005, PMID: 11317637

[ref60] Losada M. Heaphy E. (2004). The role of positivity and connectivity in the performance of business teams: a nonlinear dynamics model. Am. Behav. Sci. 47, 740–765. doi: 10.1177/0002764203260208

[ref61] Lyubomirsky S. (2008). The how of happiness: A new approach to getting the life you want. New York: Penguin.

[ref62] Lyubomirsky S. Layous K. (2013). How do simple positive activities increase well-being? Curr. Dir. Psychol. Sci. 22, 57–62. doi: 10.1177/0963721412469809

[ref63] Lyubomirsky S. Sheldon K. M. Schkade D. (2005). Pursuing happiness: the architecture of sustainable change. Rev. Gen. Psychol. 9, 111–131. doi: 10.1037/1089-2680.9.2.111

[ref64] Ma Z. Jaeger A. M. (2010). A comparative study of the influence of assertiveness on negotiation outcomes in Canada and China. Cross Cult. Manag. 17, 333–346. doi: 10.1108/13527601011086568

[ref65] Martell C. R. Dimidjian S. Herman-Dunn R. (2010). Behavioral activation for depression: A clinician’s guide. New York: Guilford Press.

[ref66] Mayer J. D. Salovey P. Caruso D. R. (2004). Emotional intelligence: theory, findings, and implications. Psychol. Inq. 15, 197–215. doi: 10.1207/s15327965pli1503_02

[ref67] Mazzucchelli T. Kane R. Rees C. (2009). Behavioral activation treatments for depression in adults: a meta-analysis and review. Clin. Psychol. Sci. Pract. 16, 383–411. doi: 10.1111/j.1468-2850.2009.01178.x, PMID: 40693313

[ref68] McCracken L. M. Badinlou F. Buhrman M. Brocki K. C. (2021). The role of psychological flexibility in the context of COVID-19: associations with depression, anxiety, and insomnia. J. Contextual Behav. Sci. 19, 28–35. doi: 10.1016/j.jcbs.2020.11.003

[ref69] McCrae R. R. Costa P. T. Jr. (1997). Personality trait structure as a human universal. Am. Psychol. 52, 509–516. doi: 10.1037//0003-066x.52.5.509, PMID: 9145021

[ref70] Mitamura T. (2018). Developing the functional assertiveness scale: measuring dimensions of objective effectiveness and pragmatic politeness. Jpn. Psychol. Res. 60, 99–110. doi: 10.1111/jpr.12185

[ref71] Neff K. (2011). Self-compassion: The proven power of being kind to yourself. New York: William Morrow.

[ref72] Neff K. D. (2023). Self-compassion: theory, method, research, and intervention. Annu. Rev. Psychol. 74, 193–218. doi: 10.1146/annurev-psych-032420-031047, PMID: 35961039

[ref73] Neff K. D. Germer C. K. (2013). A pilot study and randomized controlled trial of the mindful self-compassion program. J. Clin. Psychol. 69, 28–44. doi: 10.1002/jclp.21923, PMID: 23070875

[ref74] Norcross J. C. Goldfried M. R. (2005). Handbook of psychotherapy integration. 2nd Edn. New York: Oxford University Press.

[ref75] Omura M. Maguire J. Levett-Jones T. Stone T. E. (2017). The effectiveness of assertiveness communication training programs for healthcare professionals and students: a systematic review. Int. J. Nurs. Stud. 76, 120–128. doi: 10.1016/j.ijnurstu.2017.09.001, PMID: 28964979

[ref76] Park Y. S. Kim B. S. K. (2008). Asian and European American cultural values and communication styles among Asian American and European American college students. Cultur. Divers. Ethnic Minor. Psychol. 14, 47–56. doi: 10.1037/1099-9809.14.1.47, PMID: 18230000

[ref77] Parrott W. G. (2001). Emotions in social psychology: Essential readings. New York: Psychology Press.

[ref78] Rimm D. C. Masters J. C. (1974). Behavior therapy: Techniques and empirical findings. Oxford: Academic Press.

[ref79] Rozin P. Royzman E. B. (2001). Negativity bias, negativity dominance, and contagion. Personal. Soc. Psychol. Rev. 5, 296–320. doi: 10.1207/S15327957PSPR0504_2

[ref80] Ryan R. M. Deci E. L. (2000). Self-determination theory and the facilitation of intrinsic motivation, social development, and well-being. Am. Psychol. 55, 68–78. doi: 10.1037/0003-066X.55.1.68, PMID: 11392867

[ref81] Ryff C. D. Singer B. (1998). The contours of positive human health. Psychol. Inq. 9, 1–28. doi: 10.1207/s15327965pli0901_1

[ref82] Salter A. (1949). Conditioned reflex therapy, the direct approach to the reconstruction of personality. Oxford: Creative Age Press.

[ref83] Sarkova M. Bacikova-Sleskova M. Orosova O. Madarasova Geckova A. Katreniakova Z. Klein D.. (2013). Associations between assertiveness, psychological well-being, and self-esteem in adolescents. J. Appl. Soc. Psychol. 43, 147–154. doi: 10.1111/j.1559-1816.2012.00988.x

[ref84] Schimmack U. Diener E. (1997). Affect intensity: separating intensity and frequency in repeatedly measured affect. J. Pers. Soc. Psychol. 73, 1313–1329. doi: 10.1037/0022-3514.73.6.1313

[ref85] Schütz E. Sailer U. Al Nima A. Rosenberg P. Andersson Arntén A. C. Archer T.. (2013). The affective profiles in the USA: happiness, depression, life satisfaction, and happiness-increasing strategies. PeerJ 1:e156. doi: 10.7717/peerj.156, PMID: 24058884 PMC3775633

[ref86] Seco V. M. M. Lopes M. P. (2014). Between compassionateness and assertiveness: a trust matrix for leaders. J. Ind. Eng. Manag. 7, 622–644. doi: 10.3926/jiem.1046

[ref87] Shallcross A. J. Ford B. Q. Floerke V. A. Mauss I. B. (2013). Getting better with age: the relationship between age, acceptance, and negative affect. J. Pers. Soc. Psychol. 104, 734–749. doi: 10.1037/a0031180, PMID: 23276266 PMC3609879

[ref88] Shiffman S. Stone A. A. Hufford M. R. (2008). Ecological momentary assessment. Annu. Rev. Clin. Psychol. 4, 1–32. doi: 10.1146/annurev.clinpsy.3.022806.091415, PMID: 18509902

[ref89] Singh B. Olds T. Curtis R. Dumuid D. Virgara R. Watson A.. (2023). Effectiveness of physical activity interventions for improving depression, anxiety and distress: an overview of systematic reviews. Br. J. Sports Med. 57, 1203–1209. doi: 10.1136/bjsports-2022-106195, PMID: 36796860 PMC10579187

[ref90] Skwara A. C. King B. G. Saron C. D. (2017). “Studies of training compassion: what have we learned; what remains unknown?” in The Oxford handbook of compassion science. eds. Seppälä E. M. Simon-Thomas E. Brown S. L. Worline M. C. Cameron C. D. Doty J. R.. (New York: Oxford University Press), 219–236.

[ref91] Smith M. J. (1985). When I say no, I feel guilty: How to cope-using the skills of systematic assertive therapy. New York: Bantam.

[ref92] Speed B. C. Goldstein B. L. Goldfried M. R. (2018). Assertiveness training: a forgotten evidence-based treatment. Clin. Psychol. Sci. Pract. 25:e12216. doi: 10.1111/cpsp.12216, PMID: 40693313

[ref93] Spinrad T. L. Eisenberg N. (2017). “Compassion in children” in The Oxford handbook of compassion science. eds. Seppälä E. M. Simon-Thomas E. Brown S. L. Worline M. C. Cameron C. D. Doty J. R. (New York: Oxford University Press).

[ref94] Vohs K. D. Baumeister R. F. (2011). Handbook of self-regulation: Research, theory, and applications. 2nd Edn. New York: The Guilford Press.

[ref95] Wild J. Clark D. M. (2011). Imagery Rescripting of early traumatic memories in social phobia. Cogn. Behav. Pract. 18, 433–443. doi: 10.1016/j.cbpra.2011.03.002, PMID: 22298942 PMC3267018

[ref96] Williams J. C. Lynn S. J. (2010). Acceptance: an historical and conceptual review. Imagin. Cogn. Pers. 30, 5–56. doi: 10.2190/IC.30.1.c

[ref97] Wilson K. G. Sandoz E. K. Kitchens J. Roberts M. (2010). The valued living questionnaire: defining and measuring valued action within a behavioral framework. Psychol. Rec. 60, 249–272. doi: 10.1007/BF03395706

[ref98] Wolpe J. (1958). Psychotherapy by reciprocal inhibition. Palo Alto: Stanford University Press.

[ref99] Yoshinaga N. Nakamura Y. Tanoue H. MacLiam F. Aoishi K. Shiraishi Y. (2018). Is modified brief assertiveness training for nurses effective? A single-group study with long-term follow-up. J. Nurs. Manag. 26, 59–65. doi: 10.1111/jonm.12521, PMID: 28744987

[ref100] Zelenski J. M. Santoro M. S. Whelan D. C. (2012). Would introverts be better off if they acted more like extraverts? Exploring emotional and cognitive consequences of counterdispositional behavior. Emotion 12, 290–303. doi: 10.1037/a0025169, PMID: 21859197

